# Efficacy of multicomponent interventions on injury risk among ice and snow sports participants—a systematic review and meta-analysis

**DOI:** 10.1186/s13102-024-00921-6

**Published:** 2024-06-18

**Authors:** Zhanjiang Fan, Lanbin Min, Wenbin He, Yaorong Yang, Wen Ma, Jiayi Yao

**Affiliations:** 1 College of Educational Sciences, Xinjiang Normal University, Urumqi, 830017 China; 2 Department of Physical Education, Xinjiang Agricultural University, Urumqi, 830052 China; 3 College of Physical Education, Xinjiang Normal University, Urumqi, 830017 China; 4 No. 126 Middle School of Urumqi, Xinjiang, Urumqi, 830057 China

**Keywords:** Ice and snow sports, Injury prevention, Component-based interventions, Meta-analysis

## Abstract

**Background:**

Ice and snow sports, which are inherently high risk due to their physically demanding nature, pose significant challenges in terms of participant safety. These activities increase the likelihood of injuries, largely due to reduced bodily agility and responsiveness in cold, often unpredictable winter environments. The critical need for effective injury prevention in these sports is emphasized by the considerable impact injuries have on the health of participants, alongside the economic and social costs associated with medical and rehabilitative care. In the context of ice and snow sports environments, applying the E principles of injury prevention to evaluate intervention measures can guide the implementation of future sports safety and other health promotion intervention measures in this field. When well executed, this approach can substantially reduce both the frequency and severity of injuries, thereby significantly enhancing the safety and long-term viability of these challenging sports.

**Objective:**

The objective of this study was to rigorously assess and statistically substantiate the efficacy of diverse injury prevention strategies in ice and snow sports, aiming to bolster future safety measures with solid empirical evidence.

**Design:**

Systematic review and meta-analysis.

**Methods:**

The overarching aim of this research was to meticulously aggregate and scrutinize a broad spectrum of scholarly literature, focusing on the quantifiable efficacy of diverse, multicomponent intervention strategies in mitigating the incidence of injuries within the realm of ice and snow sports. This endeavour entailed an exhaustive extraction of data from esteemed academic databases, encompassing publications up to September 30, 2023. In pursuit of methodological excellence and analytical rigor, the study employed advanced bias assessment methodologies, notably the AMSTAR 2 and GRADE approaches, alongside sophisticated random-effects statistical modelling. This comprehensive approach was designed to ensure the utmost validity, reliability, and scholarly integrity of the study’s findings.

**Results:**

Fifteen papers, including 9 randomized controlled trials, 3 case‒control studies, and 3 cohort studies with 26,123 participants and 4,382 injuries, were analysed. The findings showed a significant reduction in injury rates through various interventions: overall injury prevention (RR = 0.50, 95% CI 0.42–0.63), educational training (RR = 0.50, 95% CI 0.34–0.73), educational videos (RR = 0.53, 95% CI 0.34–0.81), protective equipment (RR = 0.64, 95% CI 0.46–0.87), and policy changes (RR = 0.28, 95% CI 0.16–0.49). Subgroup analysis revealed potential heterogeneity in compliance (*p* = 0.347). Compared to controls, multicomponent interventions effectively reduced injury rates.

**Conclusion:**

This systematic review and meta-analysis demonstrated that multicomponent interventions significantly prevent injuries in ice and snow sports. By applying the E principles of injury prevention and constructing a framework for practical injury prevention research in ice and snow sports, we can gradually shift towards a systemic paradigm for a better understanding of the development and prevention of sports injuries. Moreover, sports injury prevention is a complex and dynamic process. Therefore, high-quality experiments in different scenarios are needed in future research to provide more reliable evidence, offer valuable and relevant prevention information for practitioners and participants, and help formulate more effective preventive measures in practice.

**Supplementary Information:**

The online version contains supplementary material available at 10.1186/s13102-024-00921-6.

## Introduction

In the scholarly realm of sports science, the term “ice and snow sports” comprehensively encapsulates a variety of activities conducted on icy and snowy terrains, such as skating, skiing, and other recreational pursuits in these environments. Empirical evidence underscores the significant role of these sports in augmenting adolescent physical health, including their instrumental contribution to mitigating psychological disorders, curbing obesity, forestalling diseases, and fortifying physical fitness [1–3]. Millions of people participate in ice and snow sports globally, mainly in countries with cold climates and relevant sports facilities, such as North America, Europe, and parts of Asia. According to a report by Snowsports Industries America, the number of winter sports participants in North America was 25.1 million in the 2019–20 season and slightly decreased to 24.6 million in the 2020–21 season. Among a diverse group of participants, only 31% are involved in winter sports, with snowboarding showing higher inclusivity, as 38% of participants come from diverse backgrounds [4]. China has successfully achieved its goal of “engaging 300 million people in ice and snow sports,” with a national participation number reaching 346 million, a participation rate of 24.56%, and a youth participation rate in ice and snow sports of 15.62%, demonstrating the widespread popularity and rapid development of ice and snow sports in China [5]. Furthermore, organizations such as the International Ski Federation (FIS), the International Skating Union (ISU), the World Curling Federation (WCF), and the International Olympic Committee (IOC) play key roles in promoting sports, establishing rules, and organizing international competitions [6]. Numerous organizations and federations have been established worldwide to promote the popularization of ice and snow sports, train athletes, and organize domestic and international competitions and events. The participation in global ice and snow sports is rapidly expanding, especially in China, where the number of participants is continually growing. Sports have expanded from specific regions and seasons to a global scope and all seasons, especially snowboarding and skiing, which are extremely popular among young people and have become fashionable physical activities [6, 7].

However, the nature of ice and snow sports includes certain risks, with part of their appeal stemming from the challenge of the natural environment and having a spirit of adventure. The potential injuries associated with these sports are a natural extension of risky behaviours. As the popularity of these sports has increased among young people, data show that from 2001 to 2023, the rates of injury to snowboarders’ heads, necks, and torsos increased by 50%; 14% of injuries occurred in adolescents, accounting for 22% of all injuries; head injuries, especially concussions, have ample epidemiological evidence indicating their significant harm, with approximately 2.5—2.9 deaths per million people in ice and snow sports. Additionally, 85% of injuries were caused by falls, 8% by collisions with others, and 5% by collisions with stationary objects. Although adolescents make up only 12% of all participants, they account for 23% of injuries; these data not only reveal the main causes of injuries but also highlight the importance of preventive measures and the cultivation of safety awareness [8–14]. The paradigm of viewing injuries as accidents, coincidental events, or random events is no longer accepted. They are understood to be predictable through causal chains of evidence and are thus considered preventable. This shift is based on the recognition that injuries can be effectively prevented by changing equipment, being aware of environmental conditions, and implementing educational interventions. Therefore, conducting targeted injury prevention interventions is crucial for reducing the risk and severity of sports injuries among participants in ice and snow sports. Moreover, the treatment of sports injuries also results in increased medical and social costs. Therefore, researching and implementing preventive interventions aimed at reducing the risk of injury is of great significance for maximizing the health benefits of participating in ice and snow sports and promoting safe participation [15].

From 1990 to 2000, research primarily focused on the effectiveness of protective gear, such as helmets [11, 16–18], wrist guards, and external joint supports [8, 9, 19, 20]. Between 2000 and 2010, the number of randomized controlled trials (RCTs) studying injury prevention in ice and snow sports and evaluating the effectiveness of protective measures nearly doubled [21]. Over the past decade, comprehensive analyses of ice and snow sports injuries have continued to increase. Recent studies have shifted their focus towards educational training programs [13, 22], educational videos [16, 23], and changes in ice and snow sports policies and regulations [10, 12–14, 24, 25] to explore the effectiveness of various intervention measures. Although previous reviews and experimental studies have evaluated the efficacy of certain specific programs [26], the diversity in content, design, target populations, and outcome reporting across different studies has limited the effective utilization of research findings. Meta-analysis can provide more comprehensive evidence in this context. Thus, our research aimed to assess the efficacy of multifaceted intervention programs in reducing injury rates and specific regional injuries, considering various age groups (children, adolescents, adults) and levels of sport participation (amateur, club, elite, mixed).

Despite extensive exploration on this topic, existing research primarily focuses on the effectiveness of individual interventions in reducing the risks associated with ice and snow sports [22–24]. It has not proposed comprehensive risk prevention strategies from an integrated perspective, which hinders educators, researchers, and ice and snow environment designers from effectively integrating research conclusions into practice. This undoubtedly increases the risk of injury for ice and snow sports participants [27]. To bridge the gap between theory and practice, researchers typically adopt comprehensive measures to ensure the well-being of individuals and communities [28, 29]. The E Principle, a commonly used framework for considering comprehensive measures in the field of injury prevention [30], integrates education, engineering, and enforcement methods. It systematically considers the effectiveness, credibility, and associated costs of intervention strategies [31, 32], promoting the translatability of interventions into applied environments [31]. Therefore, it is widely regarded as an effective guide for designing and categorizing low-risk strategies [30, 33]. In this study, to form a comprehensive prevention strategy for ice and snow sports, the E Principle was employed to shift the focus of injury responsibility from blaming the victim to recognizing the role of other stakeholders (such as organizers, policymakers, built environment designers, equipment manufacturers, and the community at large). By encouraging multi-level collaboration to develop customized risk prevention strategies, we aim to comprehensively reduce injury risks and medical costs. Additionally, using the E Principle to explore preventive measures for ice and snow sports injuries can not only help research propose comprehensive intervention recommendations [33] but also enhance the experience and well-being of ice and snow sports participants. This further promotes community well-being and advances the overall development of ice and snow sports at regional or national levels [34, 35].

Specifically, education involves providing stakeholders with educational information or training to reduce injury risks engineering involves developing products and technologies that can reduce the risk of injury, as well as engineering intervention measures to control the occurrence of injuries in ice and snow environments or designing a safer environment; enforcement includes implementing preventative rules, policies, and regulations to reduce injury risks, including the development and implementation of policies or legislation aimed at reducing or preventing hazardous behaviours; As research has deepened, the extension of the E Principle has expanded to include encouragement and evaluation as the fourth and fifth Es [36, 37]. These changes highlight the necessity of considering health promotion and providing injury prevention interventions for all community members, especially those at high risk in ice and snow sports. It also addresses the importance of formally evaluating the three Es interventions, examining the practical impact of their implementation in ice and snow sports injury prevention research. Enforcement promotes safe behaviour through incentive measures, including rewarding individuals or organizations that take safety measures or exhibit safe behaviours; and evaluation involves monitoring, assessing, and reviewing injury prevention plans and strategies to ensure their effectiveness, making adjustments, and demonstrating impact.

This study conducted a rigorous selection and analysis of literature through meta-analysis, systematically reviewing nearly 30 years of related case‒control studies, cohort studies, experimental studies, and quasiexperimental research. The analysis focused on analysing the effectiveness of multicomponent intervention measures, classifying intervention types according to the E principles and covering aspects such as educational training, educational videos, protective equipment, and changes in project policy rules to influence participant injury rates with the aim of providing more comprehensive and effective guidance for injury prevention in ice and snow sports. The study also considered various dimensions, such as age group, type of injury, level of sport, duration of intervention, and type of project, and conducted stratified subgroup analyses to explore the specific impact of intervention measures on injuries among participants in ice and snow sports under these different dimensions.

## Method

### Search strategy

Within the academic sphere of sports science, with a particular emphasis on the prevention of injuries in ice and snow sports, a comprehensive and systematic literature search was meticulously executed to collate and analyse evidence-based strategies and types of interventions. The authors of this study adhered scrupulously to the methodological protocols delineated in the Cochrane Handbook [38]. Two researchers embarked on an exhaustive and independent exploration of several prominent databases, including Google Scholar, PubMed, EMBASE, Web of Science, and Sport Discus. This search was characterized by an absence of constraints regarding publication dates, extending up to December 31, 2022. The investigative process encompassed an array of search terms intricately associated with interventions, prevention, and prophylactic measures within the realm of ice sports (such as speed skating, figure skating, ice hockey, and curling) and snow sports (encompassing skiing, snowboarding, cross-country skiing, and alpine skiing). Additionally, the search criteria included terms related to injuries, sports injuries, case studies, RCTs, and the assessment of intervention effectiveness. By employing various permutations and combinations of these keywords, the researchers ensured thorough and expansive coverage of the relevant literature. The search process was continuously updated and refined until September 30, 2023, thereby guaranteeing the inclusion of the most current and pertinent studies in this evolving field of research.

### Document recognition

**Fig. 1:**
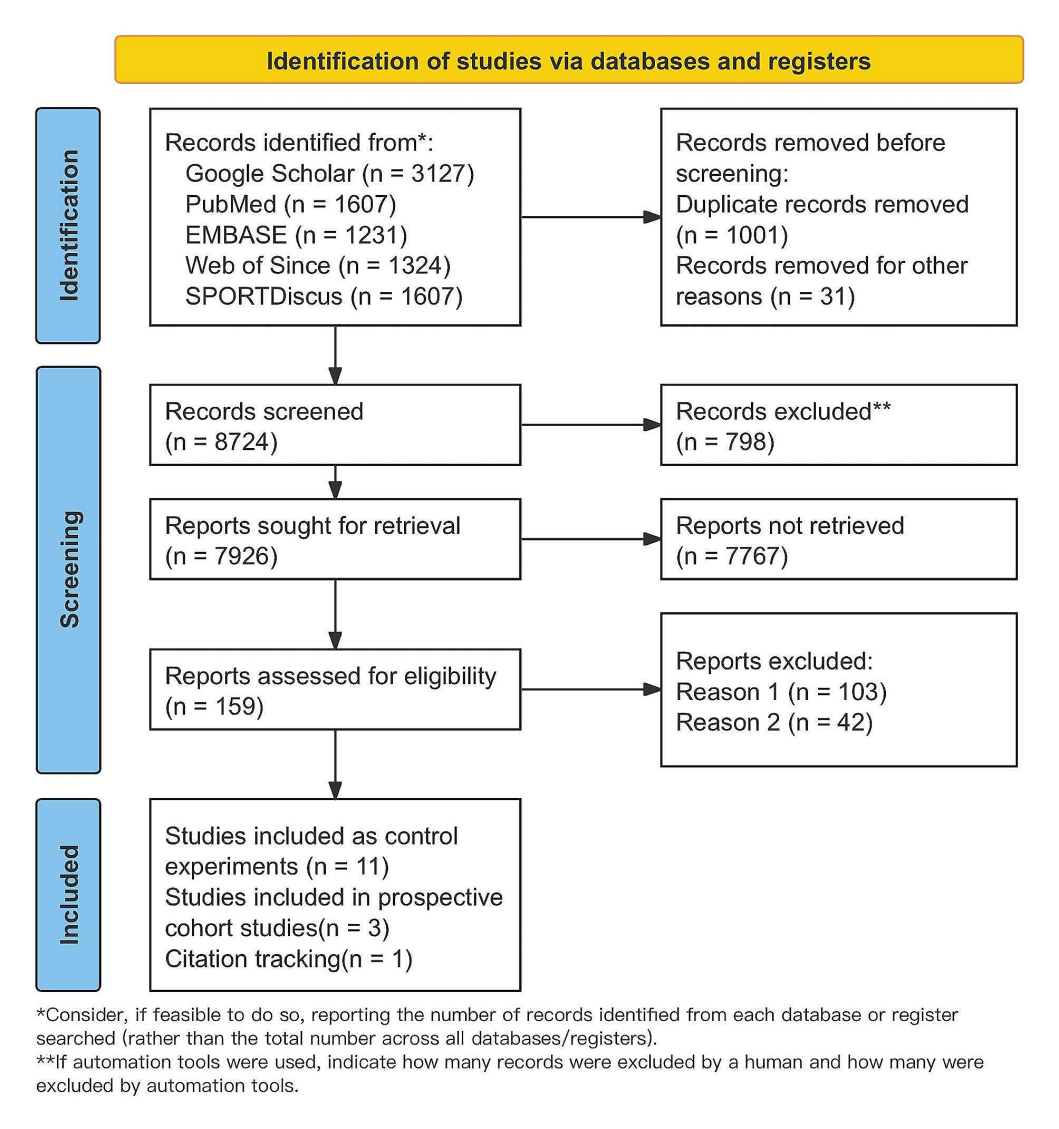
Flow chart of the study selection process

One researcher searched electronic databases and identified a total of 9,756 studies, which were subsequently saved in Zotero. After removing duplicate studies, 7,926 studies remained. An initial screening of titles and abstracts led to the exclusion of 7,767 articles, leaving 159 studies. Following a full-text review of these studies, an additional 145 studies were excluded. Additionally, a manual search of related literature and citation tracking resulted in the inclusion of one more study. Of these, 103 studies were excluded because they did not report specific injury data, and 42 studies did not meet the criteria for RCTs, case‒control studies, or prospective cohort studies. Ultimately, 15 studies were included in the meta-analysis (Fig. 1).

#### Inclusion criteria

In the meticulous process of study selection, two academically qualified researchers independently scrutinized the titles and abstracts of pertinent studies. Each study meeting the following rigorously defined inclusion criteria underwent a comprehensive full-text assessment by these researchers: (1) the study’s central theme was explicitly aligned with the prevention of injuries in the domain of ice and snow sports; (2) the methodological design of the study was structured as either a cohort study, case‒control study, or a randomized or cluster–randomized trial, ensuring a robust and scientifically sound approach; (3) the publication delineated at least one objective and quantifiable outcome, encompassing metrics such as injury rates, the total number of injuries, or the duration of the intervention, to provide measurable insights into the effectiveness of the interventions; and (4) the results presented in the study convincingly demonstrated the efficacy of the interventions in mitigating injury risks in ice and snow sports. In instances of disagreement regarding the eligibility of a specific article, the two researchers engaged in a consensus-building dialogue to resolve any discrepancies. If a consensus remained elusive, a third researcher, equipped with the requisite expertise, was enlisted to provide an adjudicative decision, thereby ensuring the integrity and scholarly rigor of the study selection process.

#### Exclusion criteria

The criteria for excluding literature were as follows: (1) risk ratios (RRs) or injury rate ratios (RRs) were not provided, or the original data could not be used to calculate the required data (for example, the use of absolute rather than relative injury rates in cohort studies); (2) only mortality rates were reported, without injury rates; (3) only the risks of injuries in ice and snow sports or the factors influencing these injuries were compared; or (4) only other data related to ice and snow sports were reported. In summary, articles that did not provide data allowing for the calculation of risk statistics or that did not provide sufficient data to calculate the injury rate RR were excluded.

### Data extraction

The study organized the interventions according to the E principles of injury prevention (education, engineering, and enforcement). The E principles of injury prevention are a commonly used framework in the field of injury prevention and are utilized for conceptualizing and categorizing effective risk reduction strategies [37. Relevant data from each study included in the full texts were extracted with the aim of evaluating the effectiveness of multicomponent interventions in preventing injuries among participants in ice and snow sports. The multicomponent intervention types mainly included the following: (1) education, which involves reducing injury risks by providing educational information or training to stakeholders, including educational training and educational videos; (2) engineering, which involves the development of products and technologies that can reduce the risk of injury, including advancements in protective equipment such as helmets and wrist guards to better prevent injuries during sports; and (3) enforcement, which includes the implementation of preventative rules, policies, and regulations to reduce the risk of injury. The injury rates for the following four types of injuries were analysed separately: (1) head injuries; (2) upper limb injuries; (3) lower limb injuries; and (4) all injuries. Table 1 provides detailed descriptions of the multicomponent interventions and injury categories.

**Table 1:** Types of multicomponent interventions and injuries

Study outcome	Description	
Educational training	ISPAInt	(1) Eccentric hamstring strength: dynamic bridging, Nordic hamstring exercise.
		(2) Leg axis stability by strengthening the external hip rotators: deep single-leg pistol squats.
		(3) Trunk stability by improving the strength and neuromuscular coordination of the trunk muscles: dynamic planking, deadbug bridging.
	NMT program	Including aerobic, strength, balance, and agility components
Educational video		(1) Educational video of ice and snow sports safety knowledge, behaviour and attitude
		(2) Ice and snow sports safety brochure
		(3) Ice and snow sports equipment use video and theoretical guidance
Policy changes		(1) Infrastructure construction
		(2) Personnel training and public participation
		(3) Rules of ice and snow sports, including competition rules, protective measures and technical standards
Protective equipment		Helmet, wrist guard, facial protector, and tooth guard
Head injuries		(1) Scalp injuries
		(2) Skull fractures
		(3) Brain injuries
Upper limb injuries		(1) Finger and palm injuries
		(2) Injuries of the elbow joint and forearm
		(3) Shoulder and upper arm injuries
Lower extremity injuries		(1) Damage to the bones, nerves, blood vessels and muscles of the lower extremities
		(2) Knee joint injuries, acute knee injuries, undefined knee injuries, ankle injuries
		(3) ACL injuries, noncontact ACL injuries
All injuries		(1) All sports injuries, all injuries
		(2) Injuries and abrasions on all parts of the body

The researchers extracted the characteristics of the participants, the type of sport, the level of sport, the duration of the intervention, and the main outcomes from each article (Table 2). The calculations for the meta-analysis were conducted using Collaboration Review Manager 5.1 software. All calculations were based on the primary outcomes of the studies. Data were analysed by calculating risk ratios (RRs), injury rate RRs, or Cox regression RRs [39]. The calculation of the injury rate RR was as follows: RR = (number of injuries in the intervention group/duration of intervention)/(number of injuries in the control group/duration of intervention). An injury rate RR > 1 was considered to indicate that the intervention effect was not significant or ineffective, while an injury rate RR < 1 was considered to indicate the effectiveness of multifaceted intervention measures in reducing injuries [40], meaning that an RR of 0.42 corresponded to a 58% reduction in injuries. The injury rate RR with a 95% confidence interval (CI) was used as the measure of effect size for analysis. The inverse variance was used as the statistical method, and the analysis was based on a random-effects model. Statistical heterogeneity was assessed using I^2^ and χ2 (Q) values; heterogeneity was considered low for I^2^ values between 25 and 50%, moderate for values between 50 and 75%, and high for values ≥ 75% [38]. Tri-tailed or bi-tailed P values < 0.05 were considered to indicate statistical significance.

**Table 2:** Characteristics of included trials and quality evaluation

Study	Intervention	Age	Session	Level	Sport	Outcome (injuries)	Compliance %	Quality grade
Schoeb et al. [22]	ISPAInt	13–15	48 weeks	Elite	Alpine skiing	All	100	High(10)
Priyambada et al. [23]	Educational video	7–16	2 weeks	Primary	Skiing	All	87	High(9)
Hagel et al. [18]	Protective equipment	< 15, 15–25, ≥ 26	24 weeks	Club	Skiing/snowboarding	Head	77	Medium(8)
Hasler et al. [11]	Protective equipment	19–20	24 weeks	Club	Skiing/snowboarding	Head	78	Medium(7)
Emery et al. [10]	NMT plan	11–15	12 weeks	Club	Ice hockey	All/limb	97	High(11)
Ytterstad et al. [19]	Education/protective equipment	0–14, ≥ 15	3 years	Club	Skiing/ice hockey	Head/all	87	Medium(8)
Cusimano et al. [20]	Educational video	11–12	16 weeks	Primary	Skiing/snowboarding	Upper limb	93	Low(7)
Machold et al. [9]	Protective equipment	11–17	1 weeks	Primary	Skiing/snowboarding	Upper limb	65	Low(7)
Jørgensen et al. [16]	Educational video	5–61	8 weeks	Mix	Alpine skiing	All	83.2	Medium(8)
Westin et al. [13]	Core stability/NAM plan	14–18	2 years	Primary	Alpine skiing	Lower limb	100	High(11)
Rønning et al. [8]	Protective equipment	10–68	12 weeks	Mix	Skiing/snowboarding	Upper limb	67	Low(7)
Kolstad et al. [14]	Policy changes/protective equipment	11–18	5 years	Club	Ice hockey	Head	87.3	High(8)
Black et al. [12]	Policy changes	11–12	2 4 weeks	Club	Ice hockey	Head/all	92	High(8)
Emery et al. [24]	Policy changes	11–12	24 weeks	Club	Ice hockey	Head	84	Medium(7)
Benson et al. [17]	Protective equipment	All	24 weeks	Club	Ice hockey	Head	78	Medium(6)

### Quality evaluation

In accordance with the recommendations of AMSTAR 2 [41], the credibility of each included experiment was assessed to categorize the studies. Two researchers evaluated each study based on fulfilment of the evaluation criteria, marking them as “yes,” “no,” or “partly yes” for some entries. Depending on the potential impact on the study results, each credibility level was judged as high, moderate, low, or very low. A study was rated as “high” if there were 0 or 1 noncritical items with flaws and “moderate” if there were more than 1 noncritical items with flaws. If there was 1 critical item with flaws with or without noncritical items with flaws, the study credibility was rated as “low.” If there was more than 1 critical item with flaws with or without noncritical items with flaws, the study credibility was rated as “very low.” The two researchers independently reviewed the credibility and resolved any discrepancies through consensus among all researchers. The quality of evidence and the strength of the recommendations were evaluated using the GRADE system [42]. Researchers considered four key elements of the articles: study design, study quality, consistency, and directness. The criteria for assigning evidence levels were as follows: (1) RCTs were rated as high-level studies; (2) observational studies as moderate-level studies; and (3) other studies as low-level studies. The level was downgraded under the following conditions: (1) poor study quality decreased the level by 1, and very poor study quality decreased the level by 2; (2) poor consistency decreased the level by 1; (3) large uncertainty in directness decreased the level by 1, and very large uncertainty decreased the level by 2; (4) unclear data reporting decreased the level by 1; and (5) high risk of bias decreased the level by 1. The level was upgraded under the following conditions: (1) consistency of two or more pieces of evidence, with significant and low risk of bias, increased the level by 1–2; (2) strong direct evidence, with significant and low risk, increased the level by 2–5, and validity of the evidence increased the level by 2; (3) each increase in the degree of evidence increased the level by 1; and (4) reduction of all potential confounding factors increased the level by 1. Publication bias was assessed through visual inspection of funnel plots and the bi-tailed Egger test [43]. Finally, the evidence was categorized into four levels: high, moderate, low, and very low. Based on this, a systematic analysis of the literature was conducted, including 9 RCTs, 3 case‒control and case‒crossover studies, and 3 prospective cohort studies, totalling 15 studies with 27 valid data points. Using 12 quality criteria adapted from Furlan [44], two researchers independently scored the methodological quality (Table 2), with the highest score being 11/12, the lowest score being 7/12, and the average score being 8/12.

### Publication bias

**Fig. 2:**
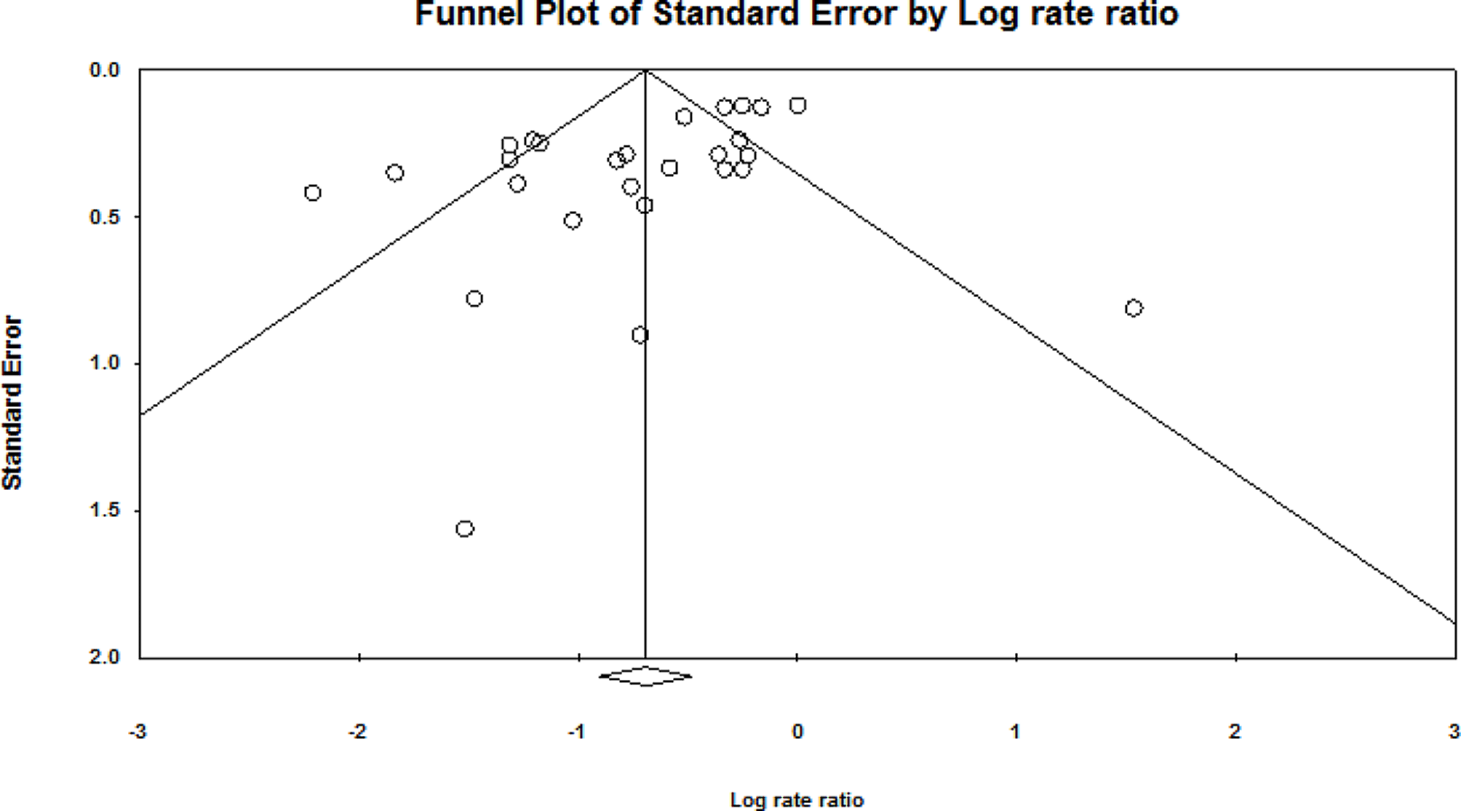
Publication bias funnel plot of the study sample

Based on the studies identified, the funnel plot (Fig. 2) showed that the effect sizes were relatively evenly clustered in the upper effective area, suggesting a symmetric distribution. To avoid a single study generating too many effect values and occupying excessive weight, potentially causing bias in the results, this study adopted a method of effect value aggregation for articles containing various conditions. If an experiment reported the effects of multiple interventions and these interventions were not the moderating variables of interest in this study, they were converted into a single effect size. Furthermore, to ensure the independence of effect values, if an experiment reported multiple test results from the same sample, CMA 3.0 was used to combine these effect values before including them in the meta-analysis. Egger’s test was used to confirm asymmetry. The larger the deviation of the intercept from zero was, the more apparent the asymmetry. If the p value of the intercept was equal to or less than 0.1, the asymmetry was considered statistically significant (intercept = -2.08, SE = 0.69, *P* = 0.003) [45]. The fail-safe number (Nfs) test criterion was an Nfs value greater than 5 *N* + 10, with N representing the number of studies. This criterion, as proposed by Rosenthal [46, 47], estimates how many unpublished and nonsignificant study samples would be needed to render the current meta-analysis results insignificant. The results showed Nfs = 926, which is greater than 5 × 27 + 10 = 145, indicating that the likelihood of a change in the results of this meta-analysis is minimal. Based on these findings, we concluded that there was no publication bias in the included studies and that the results of the meta-analysis are valid and reliable.

## Results

Through a search, review, and selection of literature, among the 15 studies included in our analysis, we focused only on initial injuries, as repeated results are likely interdependent, potentially leading to bias. According to the E principles of injury prevention, the studies included 6 education-based interventions (3 educational training and information, 3 educational videos), 7 engineering interventions (protective equipment such as helmets and wrist guards), and 2 enforcement interventions (policy and rule changes). The included studies consisted of 5 European RCTs [8, 9, 13, 16, 22], 3 Canadian RCTs [10, 20, 23], 1 prospective RCT from the United States [19], and 3 prospective cohort and case‒control studies from Canada and Switzerland [11, 12, 14]. Additionally, there were 3 prospective cohort studies from Canada [17, 18, 24]. The studies involved a total of 26,123 participants, including both males and females, with an age range covering children (0–12 years), adolescents (13–19 years), and adults (20 years and older). The number of participants in these studies varied from 69 to 6,266 [14, 20]. A total of 4,382 injuries were reported across the studies, with intervention durations ranging from 1 week to 144 weeks [11, 19]. All interventions were applied at least twice weekly in the intervention groups, while regular training was provided in the control groups. Subgroup analyses were further conducted, including analyses of variables such as age, duration of intervention, level of sport, and type of ice and snow sport. Age, sport level, intervention duration, and ice and snow sports were categorized as follows, respectively: children, adolescents, and adults; elite, club, and amateur; less than or equal to 2 weeks, 8–12 weeks, and more than 12 weeks; and skiing, snowboarding, alpine skiing, and ice hockey.

### Evaluating the efficacy of interventions

**Fig. 3:**
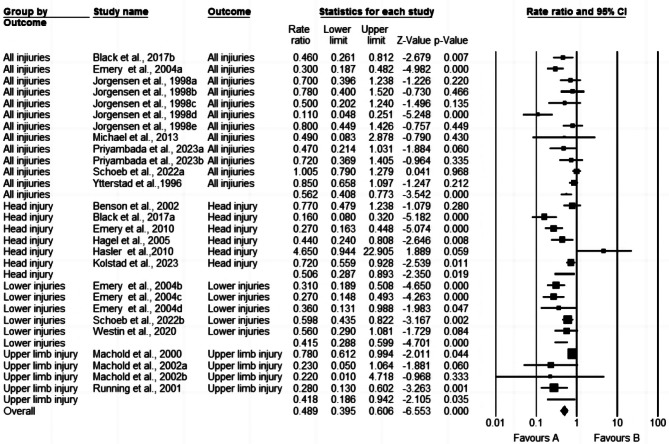
Results of the meta-analysis

In the 15 studies included, the overall impact of different interventions on the prevention of injuries in ice and snow sports showed a total injury rate ratio of 0.50 (95% CI 0.41–0.62; I^2^ = 76.56%; T^2^ = 0.195; *p* < 0.001) (Fig. 3). This indicates that compared to that in the control group, the injury rate in the intervention group was reduced by 50% (1-0.50), meaning that the injury rate in the intervention group was 50% lower than that in the control group. The 95% CI of 0.41–0.62 suggests that at the 95% confidence level, there is a 95% probability that the true injury rate RR lies between 0.41 and 0.62, indicating some degree of uncertainty about this injury rate RR. Importantly, this CI does not include 1, and a p value of < 0.001 signifies that the injury prevention effect is significant, indicating that the injury rate RR in the intervention group is significantly lower than that in the control group (Fig. 3). The Q value of 110.91 (df = 26, *P* < 0.001) highlights variability in the true effect sizes across all studies. The I^2^ of 76.56% indicates that approximately 77% of the variance observed in the effects is due to true effects. The T^2^ and T values are 0.195 and 0.442, respectively, further emphasizing the heterogeneity observed in the study results.

#### Education

**Fig. 4:**
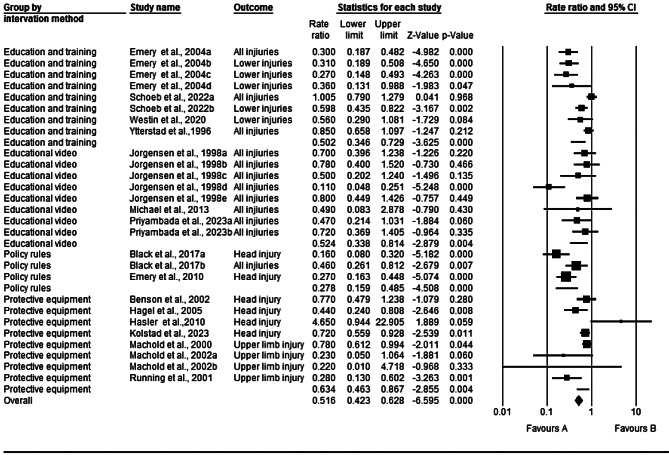
Combined effect of multicomponent interventions on the injury rate of participants

The effectiveness of educational training interventions in reducing injuries in ice and snow sports was studied in 3 experiments involving a total of 1,590 participants [10, 13, 22]. The educational training programs included the ISPAInt program and high-intensity neuromuscular training (NMT) program. The injury rate RR for ice and snow sports participants subjected to educational training interventions was 0.50 (95% CI 0.34–0.73; I^2^ = 84.61%; T^2^ = 0.223; *p* < 0.001) (Fig. 4). This indicates that educational training interventions can significantly reduce the overall injury rate. Specifically, an RR of 0.50 implies that the injury rate in groups receiving educational training interventions was 50% lower than that in groups without such interventions. The 95% CI of 0.34–0.73 suggests that there is a 95% probability that the true RR lies within this range in similar studies. The I^2^ of 84.61% indicates substantial heterogeneity in the results, warranting cautious interpretation. The T^2^ value of 0.223 suggests a small variance between different studies, which could be due to differences in study designs, sample sizes, and intervention measures. The p value < 0.001 indicates that the difference in the results is statistically significant. Overall, these results suggest that educational training interventions can reduce the overall injury rate in ice and snow sports. However, the high heterogeneity and variance should be taken into consideration.

In the three included studies on educational video interventions, comprising a total of 3,180 participants [16, 20, 23], the impact of educational video interventions on the risk of injuries among ice and snow sports participants was investigated. The injury rate RR for participants exposed to educational video interventions compared to the control group was 0.53 (95% CI 0.34–0.81; I^2^ = 62.72%; T^2^ = 0.238; *p* < 0.001) (Fig. 4). This suggests that educational video interventions can significantly reduce the overall injury rate. Specifically, the RR of 0.53 indicates that the injury rate in groups receiving educational video interventions was 47% lower than that in groups without such interventions. The 95% CI of 0.34–0.81 implies that in similar studies, there is a 95% probability that the true RR lies within this range.

The I^2^ of 62.72% indicates moderate heterogeneity in the results, while the T^2^ of 0.238 suggests a small variance between different studies. The p value < 0.001 indicates that the difference in the results is statistically significant. Overall, these results demonstrate that educational video interventions can effectively reduce the overall injury rate in ice and snow sports.

#### Engineering

In a total of 7 experiments involving 19,545 participants, the effectiveness of protective equipment in reducing injury risk among ice and snow sports participants was studied. The protective equipment mainly included helmets [11, 14, 18], wrist guards [8, 9], and facial protection, including mouth guards [17]. These participants included alpine skiers, skiers, snowboarders, and ice hockey players. In 5 experiments evaluating head and facial injuries [11, 14, 17–19], involving 13,755 participants, helmets and facial protection, including mouth guards, were found to effectively protect ice and snow athletes from head injuries. In 2 experiments assessing upper limb (wrist and shoulder) injuries, involving a total of 5,790 participants, wrist guards or external joint supports effectively protected against wrist injuries [8, 9].

Based on the effectiveness studies of protective equipment across 7 experiments, the interventions collectively reduced injuries to various body parts compared to the controls, with an injury rate RR = 0.64 (95% CI 0.46–0.87; I^2^ = 58.13%; T^2^ = 0.087; *p* < 0.01) (Fig. 4). This indicates that protective equipment interventions can significantly reduce the overall injury rate. Specifically, the RR of 0.64 suggests that the injury rate after protective equipment interventions was 36% lower than that in groups without these interventions. The 95% CI of 0.46–0.87 implies that there is a 95% probability that the true RR lies within this range in similar studies. The I^2^ of 58.13% indicates moderate heterogeneity in the results, while the T^2^ of 0.067 suggests a small variance between different studies. The p value of < 0.004 indicates that the difference in the results is statistically significant. Overall, these results demonstrate that interventions involving protective equipment can effectively reduce the overall injury rate in ice and snow sports.

#### Enforcement

Two prospective cohort studies involving a total of 1,848 participants examined the impact of policy and rule changes on injury risk among ice hockey players [10, 12]. Compared to the control group, the injury rate RR for ice hockey players subjected to interventions involving changes in policy and rules was 0.28 (95% CI 0.16–0.49; I^2^ = 63.24%; T^2^ = 0.152; *p* < 0.001) (Fig. 4). This indicates that interventions involving policy and rule changes can significantly reduce the overall injury rate. Specifically, the RR of 0.28 suggests that the injury rate after such interventions was 72% lower than that in groups without these interventions. The 95% CI of 0.16–0.49 implies that in similar studies, there is a 95% probability that the true RR lies within this range.

The I^2^ of 63.24% indicates moderate heterogeneity in the results, while the T^2^ of 0.152 suggests a small variance between different studies. The p value < 0.001 indicates that the difference in the results is statistically significant. Overall, these results demonstrate that interventions involving policy and rule changes can effectively reduce the overall injury rate in ice hockey sports.

### Subgroup analysis

The subgroup analysis primarily focused on the injury rates among ice and snow sports participants and the results of mixed-effects application of a random model across five moderating variables (Table 3). A comparison between subgroups revealed only one significant difference (*p* = 0.347). This finding offers insights for interpreting the qualitative sources within our study. On the one hand, this finding can help explain the variance among studies. On the other hand, this finding suggests that elite athletes, through years of training and competition experience, have developed good sports habits. Consequently, intervention measures may not have as significant an impact on elite athletes as they do on athletes in other groups. This lack of a significant impact on elite athletes can be attributed to their already established and effective injury prevention practices and heightened awareness and skill level in their respective sports.

**Table 3:** Subgroup analyses according to identified moderating factors

Moderator	Mixed-effects analysis of between-subgroup comparisons							Subgroup heterogeneity			
	K_*R*_	ES	95% CI	*P*_b_- value	*P*- RR-*R* (%)	Q_b_-value (df)	*P*_b_- value	Q_w_^c^- value (df)	*P*_w_^d^- value	I^2^	T^2^
Injury type						1.65 (3)	0.65				
Head	6	0.51	0.29–0.89	0.019	49			33.41 (5)	< 0.001	85.04	0.39
Upper limb	4	0.42	0.19–0.94	0.035	58			8.84 (3)	0.032	66.06	0.37
Lower limb	5	0.41	0.28–0.60	< 0.001	59			8.63 (4)	0.071	53.65	0.09
All	12	0.56	0.41–0.77	< 0.001	44			46.52 (11)	< 0.001	76.36	0.21
Age group						26.26 (3)	0.00				
Children	7	0.30	0.23–0.38	< 0.001	70			6.04 (6)	< 0.001	0.74	0.00
Adolescent	10	0.62	0.43–0.89	0.009	38			22.30 (4)	< 0.001	82.06	0.13
Adult	10	0.68	0.57–0.80	0.000	32			35.59 (9)	< 0.001	74.71	0.25
Exercise level						3.12 (3)	0.37				
Elite	2	0.78	0.47–1.30	0.347	-			6.49 (1)	0.011	84.60	0.11
Club	11	0.46	0.33–0.66	< 0.001	54			55.95 (10)	< 0.001	82.13	0.26
Primary	8	0.51	0.35–0.75	0.001	49			15.34 (7)	0.032	54.38	0.13
Mix	6	0.45	0.25–0.81	0.007	55			20.74 (5)	0.001	75.89	0.40
Duration						4.08 (2)	0.13				
≤ 2 w	5	0.70	0.54–0.91	0.007	30			4.26 (4)	0.372	6.09	0.01
8–12 w	5	0.49	0.26–0.95	0.035	51			18.03 (16)	0.001	77.81	0.43
≥ 12 w	17	0.48	0.37–0.63	< 0.001	52			86.13 (1)	< 0.001	81.42	0.23
Ice and snow sport type											
Alpine skiing	9	0.64	0.47–0.86	0.003	36	3.96 (2)	0.14	30.95 (8)	< 0.001	74.15	0.14
Skiing/snowboarding	10	0.51	0.34–0.76	0.001	49			26.48(9)	0.002	66.01	0.22
Ice hockey	8	0.38	0.25–0.57	< 0.001	62			35.10 (7)	< 0.001	80.06	0.26

*Note* Q value, total or subgroup effect value of study dispersion; K, no. of studies; R, random-effects model; ES, effect size damage rate ratio; P-RR-R, possible RR reduction; b, total between; w, total within; c, top value per moderator, indicating Q value of within-subgroup heterogeneity (the lower Q value indicates between-subgroup heterogeneity); d, top value per moderator, indicating P value of within-subgroup heterogeneity (the lower P value indicates between-subgroup heterogeneity); heterogeneity in I^2^, T^2^, and subgroups

#### Types of injuries

The subgroup analysis for types of injuries revealed the following: for head injuries, the injury rate RR was 0.51 (95% CI 0.29–0.89; I^2^ = 85.04%; T^2^ = 0.386; *p* < 0.01). This indicates a statistically significant reduction in the rate of head injuries as a result of the interventions. For upper limb injuries, the RR was 0.42 (95% CI 0.19–0.94; I^2^ = 66.06%; T^2^ = 0.374; *p* < 0.05). This suggests a significant reduction in the rate of upper limb injuries. For lower limb injuries, the RR was 0.41 (95% CI 0.28–0.60; I^2^ = 53.65%; T^2^ = 0.094; *p* < 0.001), indicating a significant reduction in lower limb injuries. For injuries to the entire body, the RR was 0.56 (95% CI 0.41–0.77; I^2^ = 76.36%; T^2^ = 0.208; *p* < 0.001), which is also statistically significant.

This study revealed that multifaceted intervention measures are more effective for preventing lower and upper limb injuries than for head and overall body injuries (RR = 0.42 vs. 0.51 and 0.56). This differential effectiveness could be related to the specific characteristics of ice and snow sports activities. For example, the nature of these sports might pose greater risks for limb injuries, making interventions targeting these areas particularly effective. The high degree of heterogeneity (I^2^ values) also suggests variability in the effect sizes across the studies, which might be attributed to differences in the types of sports, intervention methods, and participant characteristics.

#### Age group

The subgroup analysis by age group revealed the following: For children (< 12 years), the injury rate RR was 0.30 (95% CI 0.23–0.38; I^2^ = 0.74%; T^2^ = 0.001; *p* < 0.001). This indicates a significant reduction in injury rates in children as a result of the interventions. For adolescents (12–19 years), the RR was 0.62 (95% CI 0.43–0.89; I^2^ = 82.06%; T^2^ = 0.134; *p* < 0.01). This suggests a substantial but less pronounced reduction in injury rates compared to that in children. For adults (≥ 20 years), the RR was 0.68 (95% CI 0.57–0.80; I^2^ = 74.71%; T^2^ = 0.253; *p* < 0.01), indicating a significant reduction in injury rates, although the effect is less than that for children.

The analysis revealed that multifaceted intervention measures are more effective for children and adults than for adolescents. This outcome aligns with cognitive development patterns: children, who have lower self-protection awareness, are more susceptible to intervention measures and possess stronger learning capabilities and a greater willingness to accept new practices. Adults, with their rich knowledge and strong self-protection awareness, are also more receptive to interventions. Adolescents, often seeking thrill and adventure, are more likely to indulge in risky behaviour, making them more prone to accidents and injuries during sports activities. The significant heterogeneity (I^2^ values) among adolescents and adults suggests variability in the effect sizes across different studies, possibly due to variations in intervention methods, types of sports, and individual characteristics of the participants within these age groups.

#### Exercise level

The subgroup analysis by exercise level revealed the following: For elite-level athletes, the injury rate RR was 0.78 (95% CI 0.47–1.30; I^2^ = 84.6%; T^2^ = 0.114; *p* = 0.347), which is not statistically significant. This suggests that interventions have a less pronounced impact on reducing injuries among elite athletes. For club-level athletes, the RR was 0.46 (95% CI 0.33–0.66; I^2^ = 82.13%; T^2^ = 0.264; *p* < 0.001), indicating a significant reduction in injury rates at this level. For amateur-level athletes, the RR was 0.51 (95% CI 0.35–0.75; I^2^ = 54.38%; T^2^ = 0.126; *p* < 0.001), also indicating a significant reduction in injury rates. For mixed levels, the overall injury rate RR was 0.45 (95% CI 0.25–0.81; I^2^ = 75.89%; T^2^ = 0.401; *p* < 0.01), which is statistically significant.

The analysis indicates that multifaceted intervention measures are most effective for club-level participants, followed by amateur-level athletes, with no significant impact for elite-level athletes. The high heterogeneity (I^2^ values) across different levels, especially among elite and club-level athletes, suggests variability in the effect sizes, possibly due to differences in the intensity and nature of the sports activities, the athletes’ experience, and the specific types of interventions used. The lack of a significant impact on elite athletes might be attributed to their high levels of training and awareness and existing injury prevention practices. In contrast, club and amateur athletes might benefit more from interventions due to less exposure to professional training and injury prevention strategies.

#### Duration of intervention

The subgroup analysis based on the duration of the intervention revealed the following: For interventions lasting ≤ 2 weeks, the injury rate RR was 0.70 (95% CI 0.54–0.91; I^2^ = 6.09%; T^2^ = 0.009; *p* < 0.01). This indicates a significant reduction in injury rates for short-term interventions, with minimal heterogeneity among studies. For interventions lasting 8–12 weeks, the RR was 0.49 (95% CI 0.26–0.95; I^2^ = 77.81%; T^2^ = 0.428; *p* < 0.05). This suggests a more pronounced reduction in injury rates for medium-term interventions, although with a higher level of heterogeneity. For interventions lasting ≥ 12 weeks, the RR was 0.48 (95% CI 0.37–0.63; I^2^ = 81.42%; T^2^ = 0.227; *p* < 0.001). This indicates a significant reduction in injury rates for long-term interventions, again with considerable heterogeneity.

The subgroup analysis of the duration of the intervention shows that medium-term (8–12 weeks) and long-term (≥ 12 weeks) interventions are most effective, followed by short-term (≤ 2 weeks) interventions. The varying effectiveness based on duration suggests that while shorter interventions have an impact, more extended periods of intervention may be more effective in reducing injuries. The high I^2^ values for the 8- to 12-week and ≥ 12-week durations indicate substantial heterogeneity, which could be due to variations in the types of interventions implemented, the sports involved, and the specific characteristics of the participants. Despite the heterogeneity, the consistent trend across all durations underscores the overall effectiveness of intervention measures in reducing injury rates in ice and snow sports.

#### Ice and snow sports

The subgroup analysis based on the type of ice and snow sports revealed the following: For alpine skiing, the injury rate RR was 0.64 (95% CI 0.47–0.86; I^2^ = 74.15%; T^2^ = 0.136; *p* < 0.01). This indicates a significant reduction in injury rates in alpine skiing, though with considerable heterogeneity among studies. For skiing/snowboarding, the RR was 0.51 (95% CI 0.34–0.76; I^2^ = 66.01%; T^2^ = 0.223; *p* < 0.01). This suggests a significant reduction in injury rates in skiing and snowboarding, with moderate heterogeneity. For ice hockey, the RR was 0.38 (95% CI 0.25–0.57; I^2^ = 80.06%; T^2^ = 0.258; *p* < 0.001), indicating a significant reduction in injury rates and the highest effectiveness among the sports analysed, again with considerable heterogeneity.

Our analysis suggests that the efficacy of the interventions varies significantly across different ice and snow sports, with pronounced effectiveness observed in ice hockey compared to alpine skiing, skiing, and snowboarding. This differential impact may be attributed to the inherently intense physical contact and competitive ethos of ice hockey, which render it particularly amenable to the influence of policy and rule changes. The observed high levels of heterogeneity, as reflected in the I^2^ values across these sports, indicate a notable variation in the effect sizes. This variability is likely a consequence of several factors, including the distinct nature of each sport, the specific types of interventions implemented, and the unique characteristics of the participant cohorts within each sporting discipline. The analysis further reveals a significant reduction in injury rates across all examined types of ice and snow sports, emphasizing the overarching effectiveness of interventions when they are meticulously tailored to meet the specific needs and inherent risks associated with each sport. This finding underscores the critical importance of developing and implementing bespoke intervention strategies that are finely attuned to the particularities of each sport, thereby optimizing their potential to mitigate injury risks and enhance participant safety. A nuanced approach to intervention design and implementation, cognizant of the unique attributes and demands of each sport, is paramount for effectively reducing injury rates and promoting the health and safety of athletes engaged in these diverse and challenging sporting activities.

## Discussion

### Findings

This systematic review and meta-analysis, which included 9 RCTs, 3 case‒control studies, and 3 prospective cohort studies, evaluated the effectiveness of intervention measures on overall and specific injuries among participants in ice and snow sports. Excluding the influence of objective factors, such as environmental and regional factors, the measures rooted in each of the E principles of injury prevention (education, engineering, and enforcement) exhibit significant effectiveness in preventing overall and specific injuries among participants in ice and snow sports. The selected interventions based on education, engineering, and enforcement reduce the injury rates in ice and snow sports. Although these results demonstrate the potential of the principles of injury prevention, it is not possible to compare the principles between the groups, as the interventions across the groups did not include the same outcome indicators. Based on the injury rate RRs and 95% CIs, the results demonstrate that the intervention measures effectively reduce the risk of injuries among ice and snow sports participants. The analysis of the impact of multifaceted injury prevention interventions compared to control groups on overall and regional injury risks included I^2^ values, p values, RRs, T^2^ values at a significance level of *P* < 0.001, along with the certainty of all primary and secondary outcomes. Despite the significant preventive results indicated by the analysis, potential risks of bias exist. Moreover, most of the results are based on high efficacy.

The significant outcomes suggest that multifaceted interventions are effective in reducing injury risks in ice and snow sports. However, the variability in effects (indicated by I^2^ values) and the potential biases underscore the need for cautious interpretation of these findings. The high efficacy reported in most studies emphasizes the importance of such interventions in sports injury prevention but also highlights the necessity for continuous evaluation and potential refinement of these intervention strategies.

### Comparison with existing literature

The aim of this study was to assess the effectiveness of multifaceted interventions for the prevention of injuries in ice and snow sports. When analysing the injury rate ratio from this study and comparing it with values reported in previous research, our study included participants of all ages and various skill levels in ice and snow sports (elite, club, amateur, and mixed). The injury rate RR in this study was 0.50 (95% CI 0.41–0.62; I^2^ = 76.6%; T^2^ = 0.195; *p* < 0.001), indicating an approximately 50% reduction in injury risk, which is at the upper limit reported in previous systematic reviews. This finding represents a statistically significant and clinically meaningful reduction in the prevention of injuries, similar to the reductions in injury rates reported in previous systematic reviews and meta-analyses. For example, in an educational anterior cruciate ligament (ACL) injury prevention video study, Schoeb et al. found the intervention to be effective in preventing lower limb and knee joint injuries (RR = 0.665 (95% CI 0.485–0.884) *p* < 0.001, RR = 0.699 (95% CI 0.493–0.989) *p* < 0.001) [22]. Lauersen et al. indicated that physical exercise interventions can reduce the risk of acute injuries by 35.3% (RR = 0.65, 95% CI = 0.50–0.84, *p* < 0.01) [39], while Hübscher et al. reported that multiple intervention exercises effectively reduced the risk of lower limb injuries (RR = 0.61, 95% CI = 0.49–0.77, *p* < 0.01) and that balance training alone significantly reduced the risk of ankle sprains (RR = 0.64, 95% CI = 0.46–0.90, *p* < 0.01) [48].

A systematic review of early research on the prevention of sports injuries concluded that educational training had a significant impact as a prevention strategy [49]. Home-based balance training can improve static and dynamic balance and enhance postural control during movement, potentially reducing the risk of injury and possibly improving proprioception and neuromuscular control [10]. The 50% intervention effectiveness in our study further supports the benefits of educational training, particularly in reducing the risk of lower limb joint injuries. 80% of effective educational training interventions included stability, balance, or coordination components [25], and 3 experiments with educational training interventions significantly reduced the risk of sports injuries and improved physical capabilities. In previous studies, lower limb injuries, especially ACL injuries, were a prominent issue. In our study, educational training programs primarily based on proprioceptive training significantly prevented lower limb injuries, but further detailed research is needed to determine whether such training can reduce knee injuries. Additionally, compared to the control group, the intervention group showed a lower average 2-week rate for traumatic knee injuries, knee overuse injuries, and lower back overuse injuries [13, 22, 50]. Our findings corroborate Schoeb et al.‘s finding that youth skiers performing the ISPAInt program weekly 0.8 ± 0.6 times had a lower absolute incidence of traumatic and overuse injuries. Westin et al. reported a 45% reduction in ACL injury rates among U18 skiers [13]. Therefore, high-quality implementation should be based on a partnership between program developers (researchers) and participants. Two experiments studied the impact of high-intensity NMT programs on lower limb injuries [10, 13]. Emery et al.‘s study showed protective effects for all injuries (RR = 0.30, 95% CI, 0.19–0.49), lower limb injuries (RR = 0.31, 95% CI, 0.19–0.51), ankle sprains (RR = 0.27, 95% CI, 0.15–0.50), and knee twists (RR = 0.36, 95% CI, 0.13–0.98). Emery et al.‘s RCT showed that adolescents who underwent 12 weeks of high-intensity NMT had significantly lower risks of sports and muscle injuries than did those in the control group, with an RR of 0.82 (95% CI 0.71–0.94; 95% CI 0.58–1.15), although the difference was not significant [26]. Rahnema et al.‘s quasiexperimental study revealed significant correlations between improvements in balance and agility following 8 weeks of regular training and thrice-weekly core stability training among professional speed skaters (*p* < 0.05), indicating a positive impact on dynamic balance and agility [51, 52]. This reflects the overall trend in injury prevention research, where external risk factors are uncontrollable, but factors such as cognitive level, physical fitness, muscle strength, sports skills, and abilities can be altered through various combinations of educational interventions. Similarly, NMT is included in educational training interventions. According to the review, NMT is believed to have beneficial effects on joint position sense, stability, and reflexes. NMT is a cost-effective training method that can effectively reduce injury risk without equipment. ISPAInt interventions and strength NMT can effectively reduce overall injuries in ice and snow athletes [10, 22]. Interventional experimental studies aimed at strengthening power and improving NMT have not been widely conducted in ice and snow sports. Instead, strength training and NMT have been successfully applied as parts of multifaceted interventions, almost all of which include elements of strength, neuromuscular, balance, and coordination training. This comprehensive educational training program intervention might be the sum of all effective methods. It is challenging to pinpoint which part of the training intervention is the most effective component and which part has no impact on injury risk [49]. A combination effect might occur, but effective prevention must be based on high compliance with the injury prevention program by participants and organizers [53].

Educational video interventions have been rated as 65% effective [54, 55], which is very similar to the findings of our study. Although our results carry potential biases, our research was based on participants of all ages and varying skill levels and considered differences among subgroups. Our analysis suggests that this type of intervention has significant potential for preventing sports injuries, warranting further research into the effectiveness of educational video interventions. Additionally, the design of broader educational video intervention programs will inevitably increase with greater application, potentially leading to reduced compliance. Our study indicates that efficacy research for multifaceted intervention measures must be based on high-quality RCTs, with further research in randomized trials remaining crucial. For instance, Jørgensen et al. found that showing a 45-minute educational video during long bus trips to ski resorts for beginners, including basic skills and safety requirements, equipment checks, and helmet use, effectively reduced injury risks, especially for collisions and falls [16]. Ytterstad et al. provided past injury information and technical and safety tips to ski club members through brochures and educational videos, significantly reducing skiing injuries [19]. Using standardized assessment tools to evaluate injury rates, Priyambada et al. found that the injury risk in the intervention group was similar to that in the control group, with an injury rate of 22.95/100 (95% CI: 17.63–28.26) in the intervention video group and 23.31/100 (95% CI: 16.75–29.87) in the control group. They suggested understanding risky behaviours to optimize the promotion of safe practices and prevent injuries and appropriately incorporating them into injury prevention strategies [23]. Educational videos were found to effectively increase injury awareness and safety prevention knowledge among children and adolescent skiers, similar to the findings reported by Jørgensen et al. Intervention with snowboarding safety videos and manuals increased safety injury knowledge by 13.6% among Canadian 7th-grade (11–12 years old) students, which is a critical first step as children and adolescents face risks of preventable injuries; additionally, early learning of safety strategies could lead to lifelong safety compliance [56].

Protective equipment is widely used to prevent injuries among participants in ice and snow sports, but its effectiveness varies. Early review studies have shown that helmet use by skiers can effectively reduce the risk of head injuries [9, 11, 16, 19, 57, 58] and may also help reduce neck and other injuries [11, 40], but it could also potentially increase the risk of head or neck injuries [59]. In ice and snow sports, a mandatory policy of wearing wrist guards implemented among middle school students (12–16 years) significantly decreased wrist injury rates [60]. However, using wrist guards may increase the risk of injuries to the elbow, upper arm, and shoulder while reducing the risk to the hand, wrist, and forearm [18], possibly due to the transmission of impact forces along the kinetic chain of the limb.

In our study, 5 out of 7 experiments supported the use of protective equipment (such as helmets, face shields, and mouthguards) to effectively prevent head injuries [11, 14, 18, 19, 61]. These participants included alpine skiers, skiers, snowboarders, and ice hockey players. For instance, three case‒control studies reported a reduced risk of head injuries in participants wearing helmets (reductions of 29%, 60%, and 15%, respectively) [18], and a large study of 1,033 professional ice hockey players revealed that athletes wearing mouthguards had significantly less severe symptoms than those who did not (*p* < 0.01) [17]. In two experiments assessing upper limb (wrist and shoulder) injuries, wrist guards or external joint supports effectively protected ice and snow sports participants from wrist injuries [8, 9]. Wrist injuries are common among skiers; hence, wrist protectors with specific designs have been developed and shown significant protective effects [9]. Rønning et al., by randomly assigning snowboarders to an intervention or control group, found a significant reduction in wrist injuries in the group using wrist guards. While the results show significant preventive effects, potential risks also exist. An undisputed fact is that almost all ice and snow sports venues require participants to wear protective equipment, corroborating our findings. Further data are needed to understand which aspects of protective equipment may carry potential risks.

Additionally, numerous studies on policy and rule changes have confirmed their effectiveness in preventing injuries among ice hockey players [12, 24, 56, 62, 63]. For instance, in several studies evaluating the impact of prohibiting body checking, both injuries and penalties decreased, along with a reduction in injury rates. Regnier et al. found that in leagues where body checking was allowed (ages 11–12), players faced a greater risk of severe injuries. In Ontario and Quebec, in leagues allowing body checking (ages 14–15), players had greater injury rates than those in leagues where body checking was not permitted. The increased injury risk in leagues allowing body checking suggests that changes to body checking rules can be beneficial for protecting players. From a player development perspective, introducing body checking at an earlier age can be highly beneficial for the growth of adolescents, eliminating the career risks brought about by injuries [62]. Black et al. noted that in nonelite Canadian ice hockey games [12], abolition of the body-checking policy led to a relative reduction of 50% in injury rates and 64% in concussion rates among Alberta’s 11- and 12-year-old ice hockey players [14], with a threefold decrease in injury and concussion risks [12]. Slaney noted that mandatory wrist guard wearing in schools can effectively reduce the risk of upper limb fractures. However, the effectiveness of implementing these policies outside the school environment remains unknown [60].

Therefore, changes in policies and rules fundamentally alter the culture of a sport while maintaining the common interests of stakeholders. These findings corroborate those of our study, suggesting that policy and rule interventions have effective potential for preventing injuries in ice and snow sports. Therefore, it is necessary to develop sports rules and policies encompassing various dimensions to ensure the common interests of stakeholders, which is crucial for ensuring the sustainable development and nurturing of talent in these sports.

### **The E**’s **of ice and snow sports injury prevention**

Based on this meta-analysis, which assessed the strength of the evidence for the efficacy of intervention measures, future research needs to consider the “optimal” study design and appropriate analytical tools to achieve this goal. The practical research framework for injury prevention in ice and snow sports refers to a series of steps or measures to prevent injuries during these activities. This framework is built upon an understanding of the context for implementing injury prevention and can provide an evidence base for the effective implementation of interventions, which is essential for advancing injury prevention in ice and snow sports. This task may be challenging and requires addressing many challenges; however, all these challenges are profoundly meaningful. With advances in traditional scientific and analytical methods in sports injury research, we are gradually moving towards a systematic paradigm to better understand the development and prevention of sports injuries [33, 64, 65]. Research on preventing injuries in ice and snow sports should include key information, regardless of the design, including the reasons for employing or not employing certain measures. Indeed, adopting an evidence-based framework for injury prevention in ice and snow sports can result in improved efficacy in real-world settings.

#### Risk identification

Before initiating any preventive measures, it is crucial to identify the risks and types of injuries that participants may encounter [32]. This includes understanding the characteristics of ice and snow sports, the conditions of ice and snow sports environments, the skill levels of the participants, and other factors that may lead to injuries.

#### Risk assessment

After identifying potential risks, the next step is to assess the severity and likelihood of these risks [31, 32]. This can be done by analysing past injury data, the physical condition of the participants, and the condition of the equipment, among other factors.

#### Risk management

Based on the results of the risk assessment, measures that could reduce the risk or severity of sports injuries should be formulated [32]. While some characteristics, such as age, sex, or a history of injuries, have been shown to affect risk and recovery time, understanding these nonmodifiable risk factors is crucial for guiding interventions and strategies. This may include technical training, using appropriate protective gear, and improving the sports environment, among other measures.

#### Multicomponent interventions

Providing athletes, coaches, and stakeholders with the necessary education, engineering, enforcement, and encouragement measures will enhance their awareness [64]and ability to prevent injuries in ice and snow sports. This includes proper sports techniques, methods of using protective equipment, policy support and encouragement measures, and first aid measures in the event of an accident.

#### Implementation and execution

Evidence-based prevention strategies should be applied to reduce the risk of injuries [31]. Preventive measures should be implemented to ensure that all participants adhere to relevant safety guidelines and procedures.

#### Monitoring and evaluation

Specific methods should be developed for planning, analysing, and evaluating the effectiveness of intervention measures [31, 32, 64]. Regular monitoring and evaluation of the effects of preventive measures are critical for making necessary adjustments. This involves collecting and analysing data on the interventions, feedback, and participant satisfaction. This key step of the injury prevention process is very important yet challenging.

This evidence-based framework, through a systematic approach, aims to reduce the risk of injuries in ice and snow sports, ensuring the safety of participants. Implementing this framework requires the collective effort of all participants, including athletes, coaches, organizers, and stakeholders. Monitoring and evaluation are often overlooked. However, evaluating whether intervention measures have successfully reduced the risk of injuries is crucial. This typically means revisiting risk identification to reassess the extent of injuries [32, 66]. Ideally, this evaluation process should be a continuous part of the risk identification process, not a completely separate step. Note that these steps do not have to be completed in sequence. Due to often limited resources, adaptation to real-world settings is necessary for the effective use of time and effort. Therefore, this research provides evidence-based strategies and interventions for reducing injury risks and promote health in participants of ice and snow sports.

### Strengths and limitations

In the scholarly assessment of literature quality within our study, we adhered to the AMSTAR 2 criteria, a rigorous standard for evaluating research bias. According to this framework, a study is deemed to exhibit a low risk of bias if it fulfils at least 7 out of 7 critical items without major methodological shortcomings. Conversely, studies scoring below 5 or those with significant flaws are classified as having a high risk for bias. In our meta-analysis, only 7 studies were judged as low risk, with 4 rated as moderate risk, 3 as high risk, and 1 as very low risk. This categorization highlights the methodological diversity and potential issues of internal validity in the sampled studies.

Moreover, the issue of external validity is salient. The included studies encompassed a broad spectrum of participants across various age groups and skill levels, potentially limiting the extrapolation of our findings to elite athletic contexts. This limitation underscores the need for future empirical investigations in this area. Notably, the incorporation of case‒control and prospective cohort studies may have attenuated the overall robustness of the evidence. Our subgroup analyses, despite being meticulously conducted, involved variability in intervention approaches, study designs, and participant demographics. Our goal was to collect as much reliable evidence as possible for the prevention of injuries in ice and snow sports through researching practical preventive strategies. This endeavour entailed synthesizing a diverse corpus of data and confronting the inherent complexities of integrating various methodologies and participant cohorts. While this strategy yields an expansive understanding, it also necessitates a nuanced interpretation of the results, considering the varied degrees of bias and potential constraints in generalizing outcomes across different populations and sporting disciplines.

The dynamic and multifaceted nature of sports injury prevention mandates adaptability to real-world contexts and diverse frameworks [67]. Current research on ice and snow sports injury prevention predominantly addresses scenario-specific solutions, yet there is a burgeoning need to reinforce practical applications. Given the unique and evolving nature of implementation scenarios, strategies tailored to a singular context may not suffice. Future research should pivot towards elucidating the underpinnings of effective methods in dynamic scenarios and identifying key elements that enhance the impact of these interventions. With an emphasis on process-oriented approaches over singular solutions, the focus should be on the comprehensive efficacy of intervention programs and their implementation trajectories. A practical, scalable, and adaptable intervention program, when applied with creativity and flexibility, can provide a robust theoretical and practical foundation for designing and implementing context-specific strategies [68]. In addition to utilizing the pillars of the three “E”s for strategic interventions, other considerations of these efforts should also focus on the “fourth and fifth E”s, including encouragement, monitoring, and evaluation [64], to provide coaches, practitioners, and participants with valuable and relevant data in order to help them develop more effective prevention measures in practice. While our study primarily explored the efficacy of multifaceted intervention measures, future research should explore the intrinsic mechanisms and situational applicability of these interventions and concentrate on the intricacies of the injury prevention process. Such an approach will enable the customization of interventions to specific contexts, thereby enhancing their overall effectiveness and applicability.

## Conclusion

This study included RCTs, case‒control studies and prospective cohort studies on the prevention of injuries in ice and snow sports. By synthesizing 27 data samples from 15 studies, various intervention measures were found to effectively reduce the injury risk among ice and snow sports participants by 50% (RR = 0.50, 95% CI 0.41–0.62). Multifaceted intervention measures reduced the risk by 48% (RR = 0.52, 95% CI 0.42–0.63), with education, including training, reducing the risk by 50% (RR = 0.50, 95% CI 0.34–0.73), educational videos reducing the risk by 47% (RR = 0.53, 95% CI 0.34–0.81); engineering, including protective equipment, reducing the risk by 36% (RR = 0.64, 95% CI 0.46–0.87), and enforcement, including policy and rule changes, reducing the risk by 72% (RR = 0.28, 95% CI 0.16–0.49). A decrease in injury risk contributes to reducing the subsequent economic costs and social cost‒benefit ratio of treatment.

Recognizing that sports injuries constitute a formidable impediment to the enthusiasm and well-being of participants in ice and snow sports and considering their substantial economic implications, our study’s findings are firmly rooted in evidence-based research. The prevalence of injuries in these sports settings can be effectively mitigated, at least partially, through strategic intervention measures such as comprehensive educational training programs. The proactive promotion and implementation of these evidence-based interventions stand to confer significant additional benefits. Thus, injuries in ice and snow sports can to some extent be prevented through implementation of the E principles, constituting a shift towards a systematic paradigm with greater benefits through vigorous promotion in practice. Therefore, it is essential to promote an evidence-based framework for research on injury prevention in ice and snow sports; participants in ice and snow sports will benefit from such easy-to-implement and cost-effective injury prevention frameworks. The future of these sports is inextricably linked to the development and adoption of interventions that are not only easy to implement but also cost-effective. Such injury prevention programs are crucial for safeguarding the health and fostering the continued participation of athletes, thereby ensuring the sustainable growth and vitality of these sporting disciplines. The integration of these measures into standard practice will not only enhance the safety and enjoyment of participants but also contribute to the overall economic efficiency of these sports by reducing the costs associated with sports-related injuries.

### Electronic supplementary material

Below is the link to the electronic supplementary material.

Supplementary Material 1

## Data Availability

Data is provided within the manuscript or supplementary information files.
