# Supplementary material for: Efficacy of multicomponent interventions on injury risk among ice and snow sports participants—a systematic review and meta-analysis
Source: BMC Sports Sci Med Rehabil. 2024 Jun 18;16:135. doi: 10.1186/s13102-024-00921-6 (PMC11186188; doi:10.1186/s13102-024-00921-6)
Supplement: Supplementary file 1 — Supplementary Material 1 [file 13102_2024_921_MOESM1_ESM.docx]

Supplementary Data 1：**Characteristics of included trials**

| **Study** | **Outcome** | **RR** | **Efficte siae(95%)** | **Age** | **Level** | **Sport** | **Intervention** | **Session** | **Test type** | **Sex** |
| --- | --- | --- | --- | --- | --- | --- | --- | --- | --- | --- |
| Benson, B. W., Rose, M. S., & Meeuwisse, W. H. (2002). The impact of face shield use on concussions in ice hockey: a multivariate analysis. | Head injury | 0.77 | 0.48-1.24  0.95 | <12y；12-19y；≥20y | Club | Ice hockey | Protective equipment | ≥12w | Randomized controlled trial | m1010/w1338 |
| Black, A. M., Hagel, B. E., Palacios-Derflingher, L., Schneider, K. J., & Emery, C. A. (2017)a. The risk of injury associated with body checking among Pee Wee ice hockey players: an evaluation of Hockey Canada’s national body checking policy change. | Head injury | 0.16 | 0.08-0.32  0.95 | <12y | Club | Ice hockey | Policy rules | ≥12w | Case-control and case-crossing study | m1457/w1837 |
| Black, A. M., Hagel, B. E., Palacios-Derflingher, L., Schneider, K. J., & Emery, C. A. (2017)b. The risk of injury associated with body checking among Pee Wee ice hockey players: an evaluation of Hockey Canada’s national body checking policy change. | All injiuries | 0.46 | 0.26-0.81  0.95 | <12y | Club | Ice hockey | Policy rules | ≥12w | Case-control and case-crossing study | m1457/w1837 |
| Emery, C. A., Cassidy, J. D., Klassen, T. P., Rosychuk, R. J., & Rowe, B. H. (2004)a. (2) EFFECTIVENESS OF A PROPRIOCEPTIVE BALANCE TRAINING PROGRAM IN HEALTHY ADOLESCENTS: A CLUSTER RANDOMIZED CONTROLLED TRIAL. | All injiuries | 0.3 | 0.19-0.49  0.95 | 12-19y | Primary | Skiing / snowboarding | Education and training | ≥12w | Case-control and case-crossing study | m321/w238 |
| Emery, C. A., Cassidy, J. D., Klassen, T. P., Rosychuk, R. J., & Rowe, B. H. (2004)b. (2) EFFECTIVENESS OF A PROPRIOCEPTIVE BALANCE TRAINING PROGRAM IN HEALTHY ADOLESCENTS: A CLUSTER RANDOMIZED CONTROLLED TRIAL. | Lower injuries | 0.31 | 0.19-0.51  0.95 | <12y | Club | Ice hockey | Education and training | ≥12w | Case-control and case-crossing study | m/w |
| Emery, C. A., Cassidy, J. D., Klassen, T. P., Rosychuk, R. J., & Rowe, B. H. (2004)c. (2) EFFECTIVENESS OF A PROPRIOCEPTIVE BALANCE TRAINING PROGRAM IN HEALTHY ADOLESCENTS: A CLUSTER RANDOMIZED CONTROLLED TRIAL. | Lower injuries | 0.27 | 0.15-0.50  0.95 | <12y | Club | Ice hockey | Education and training | ≥12w | Case-control and case-crossing study | m/w |
| Emery, C. A., Cassidy, J. D., Klassen, T. P., Rosychuk, R. J., & Rowe, B. H. (2004)d. (2) EFFECTIVENESS OF A PROPRIOCEPTIVE BALANCE TRAINING PROGRAM IN HEALTHY ADOLESCENTS: A CLUSTER RANDOMIZED CONTROLLED TRIAL. | Lower injuries | 0.36 | 0.13-0.98  0.95 | <12y | Club | Ice hockey | Education and training | ≥12w | Case-control and case-crossing study | m/w |
| Emery, C. A., Kang, J., Shrier, I., Goulet, C., Hagel, B. E., Benson, B. W., ... & Meeuwisse, W. H. (2010). Risk of injury associated with body checking among youth ice hockey players | Head injury | 0.27 | 0.16-0.44  0.95 | <12y | Club | Ice hockey | Policy rules | ≥12w | Case-control and case-crossing study | m/w |
| Hagel, B. E., Pless, I. B., Goulet, C., Platt, R. W., & Robitaille, Y. (2005). Effectiveness of helmets in skiers and snowboarders: case-control and case crossover study. | Head injury | 0.44 | 0.24-0.81  0.95 | <12y；12-19y；≥20y | Club | Skiing / snowboarding | Protective equipment | ≥12w | Case-control and case-crossing study | m/w |
| Hasler, R. M., Berov, S., Benneker, L., Dubler, S., Spycher, J., Heim, D., ... & Exadaktylos, A. K. (2010). Are there risk factors for snowboard injuries? A case-control multicentre study of 559 snowboarders. | Head injury | 4.65 | 0.95-23.1  0.95 | <12y；12-19y；≥20y | Club | Skiing / snowboarding | Protective equipment | ≥12w | Case-control and case-crossing study | m/w |
| Jørgensen, U., Fredensborg, T., Haraszuk, J. P., & Crone, K. L. (1998)a. Reduction of injuries in downhill skiing by use of an instructional ski-video: a prospective randomised intervention study. | All injiuries | 0.7 | 0.39-1.22  0.95 | <12y；12-19y；≥20y | Mix | Alpine skiing | Educational video | 8-12w | Case study | m/w |
| Jørgensen, U., Fredensborg, T., Haraszuk, J. P., & Crone, K. L. (1998)b. Reduction of injuries in downhill skiing by use of an instructional ski-video: a prospective randomised intervention study. | All injiuries | 0.78 | 0.4-1.52  0.95 | <12y；12-19y；≥20y | Mix | Alpine skiing | Educational video | 8-12w | Case-control and case-crossing study | m/w |
| Jørgensen, U., Fredensborg, T., Haraszuk, J. P., & Crone, K. L. (1998)c. Reduction of injuries in downhill skiing by use of an instructional ski-video: a prospective randomised intervention study. | All injiuries | 0.5 | 0.20-1.23  0.95 | <12y；12-19y；≥20y | Mix | Alpine skiing | Educational video | 8-12w | Randomized controlled trial | m/w |
| Jørgensen, U., Fredensborg, T., Haraszuk, J. P., & Crone, K. L. (1998)d. Reduction of injuries in downhill skiing by use of an instructional ski-video: a prospective randomised intervention study. | All injiuries | 0.11 | 0.05-0.26  0.95 | <12y；12-19y；≥20y | Mix | Alpine skiing | Educational video | 8-12w | Randomized controlled trial | m/w |
| Jørgensen, U., Fredensborg, T., Haraszuk, J. P., & Crone, K. L. (1998)e. Reduction of injuries in downhill skiing by use of an instructional ski-video: a prospective randomised intervention study. | All injiuries | 0.8 | 0.45-1.43  0.95 | <12y；12-19y；≥20y | Mix | Alpine skiing | Educational video | 8-12w | Randomized controlled trial | m/w |
| Kolstad, A. T., Eliason, P. H., Galarneau, J. M., Black, A. M., Hagel, B. E., & Emery, C. A. (2023)b. Protective equipment in youth ice hockey: are mouthguards and helmet age relevant to concussion risk?. | Head injury | 0.72 | 0.56-0.93  0.95 | 12-19y | Club | Ice hockey | Protective equipment | ≥12w | Randomized controlled trial | m/w |
| Machold, W., Kwasny, O., Eisenhardt, P., Kolonja, A., Bauer, E., Lehr, S., ... & Fuchs, M. (2002)b. Reduction of severe wrist injuries in snowboarding by an optimized wrist protection device: a prospective randomized trial. | Upper limb injury | 0.78 | 0.610.99  0.95 | <12y；12-19y | Primary | Skiing / snowboarding | Protective equipment | ≤2w | Randomized controlled trial | m/w |
| Machold, W., Kwasny, O., Eisenhardt, P., Kolonja, A., Bauer, E., Lehr, S., ... & Fuchs, M. (2002)a. Reduction of severe wrist injuries in snowboarding by an optimized wrist protection device: a prospective randomized trial. | Upper limb injury | 0.23 | 0.05-1.07  0.95 | <12y；12-19y | Primary | Skiing / snowboarding | Protective equipment | ≤2w | Randomized controlled trial | m435/w286 |
| Machold, W., Kwasny, O., Eisenhardt, P., Kolonja, A., Bauer, E., Lehr, S., ... & Fuchs, M. (2002)c. Reduction of severe wrist injuries in snowboarding by an optimized wrist protection device: a prospective randomized trial. | Upper limb injury | 0.22 | 0.01-4.6  0.95 | <12y；12-19y | Primary | Skiing / snowboarding | Protective equipment | ≤2w | Randomized controlled trial | m435/w286 |
| Cusimano, M., Luong, W. P., Faress, A., Leroux, T., & Russell, K. (2013)a. Evaluation of a ski and snowboard injury prevention program. | All injiuries | 0.49 | 0.08-2.76  0.95 | <12y | Primary | Skiing / snowboarding | Educational video | ≥12w | Randomized controlled trial | m/w |
| Priyambada Mitra, T., Djerboua, M., Mahmood, S., Nettel-Aguirre, A., Caird, J. K., Emery, C., ... & Russell, K. (2023)a. Effectiveness of an injury prevention video on risky behaviours in youth snow sports: A randomized controlled trial. | All injiuries | 0.47 | 0.21-1.01  0.95 | <12y；12-19y | Primary | Skiing / snowboarding | Educational video | ≤2w | Randomized controlled trial | m1010/w1338 |
| Priyambada Mitra, T., Djerboua, M., Mahmood, S., Nettel-Aguirre, A., Caird, J. K., Emery, C., ... & Russell, K. (2023)b. Effectiveness of an injury prevention video on risky behaviours in youth snow sports: A randomized controlled trial. | All injiuries | 0.72 | 0.36-1.37  0.95 | <12y；12-19y | Primary | Skiing / snowboarding | Educational video | ≤2w | Randomized controlled trial | m1010/w1338 |
| Rønning, R., Rønning, I., Gerner, T., & Engebretsen, L. (2001). The efficacy of wrist protectors in preventing snowboarding injuries. | Upper limb injury | 0.28 | 0.13-0.60  0.95 | <12y；12-19y；≥20y | Mix | Skiing / snowboarding | Protective equipment | ≥12w |  | m/w |
| Schoeb, T., Fröhlich, S., Frey, W. O., Verhagen, E., Farshad, M., & Spörri, J. (2022)a. The ISPAInt injury prevention programme for youth competitive alpine skiers: A controlled 12-month experimental study in a real-world training setting. | All injiuries | 1.005 | 0.79-1.28  0.95 | 12-19y | Elite | Alpine skiing | Education and training | ≥12w | Randomized controlled trial | m58/w71 |
| Machold, W., Kwasny, O., Eisenhardt, P., Kolonja, A., Bauer, E., Lehr, S., ... & Fuchs, M. (2002). Reduction of severe wrist injuries in snowboarding by an optimized wrist protection device: a prospective randomized trial. | Lower injuries | 0.598 | 0.43-0.82  0.95 | 12-19y | Elite | Alpine skiing | Education and training | ≥12w | Randomized controlled trial | m58/w71 |
| Westin, M., Harringe, M. L., Engström, B., Alricsson, M., & Werner, S. (2020). Prevention of anterior cruciate ligament injuries in competitive adolescent alpine skiers. | Lower injuries | 0.56 | 0.29-1.08  0.95 | 12-19y | Primary | Alpine skiing | Education and training | ≥12w | Randomized controlled trial | m363/w373 |
| Ytterstad, B. (1996). The Harstad injury prevention study: the epidemiology of sports injuries. An 8 year study. | All injiuries | 0.85 | 0.66-1.10  0.95 | <12y；12-19y；≥20y | Club | Alpine skiing | Education and training | ≥12w | Prospective cohort study | m/w |
